# Remimazolam compared with propofol, dexmedetomidine, and midazolam for adult sedation in flexible bronchoscopy: a systematic review and meta-analysis

**DOI:** 10.1016/j.bjane.2026.844729

**Published:** 2026-01-24

**Authors:** Luiz Fábio Silva Ribeiro, Lucas Rezende de Freitas, Tauãna Terra Cordeiro de Oliveira, Laiz Gomes Carneiro Novaes, Rafael Arsky Lombardi

**Affiliations:** a Universidade Federal de São João del-Rei, Department of Medicine, Divinópolis, MG, Brazil; b Universidade Federal de Juiz de Fora, Department of Medicine, Juiz de Fora, MG, Brazil; c Universidade Estácio de Sá, Department of Medicine, Campus Vista Carioca, Rio de Janeiro, RJ, Brazil; d University of Nebraska Medical Center, Department of Anesthesiology, Omaha, United States of America

**Keywords:** Dexmedetomidine, Flexible bronchoscopy, Midazolam, Propofol, Remimazolam, Sedation

## Abstract

**Background:**

Remimazolam, a short-acting benzodiazepine, has emerged as a potential safer alternative for sedation in Flexible Bronchoscopy (FB). This meta-analysis compares its efficacy and safety with Propofol, Dexmedetomidine, and Midazolam in adult patients undergoing FB.

**Methods:**

PubMed, Embase, and Cochrane databases were searched on July 17, 2025, for trials comparing Remimazolam with other sedatives. Primary outcomes included hypotension, bradycardia, and intraprocedural opioid consumption; secondary outcomes were hypoxia, respiratory depression, patient satisfaction, induction time, and recovery time. Pooled Risk Ratios (RR), Mean Differences (MD), and Standardized Mean Differences (SMD) were calculated using a random-effects model in R (4.4.0). Risk of bias was assessed using the RoB2 tool, and subgroup analyses were conducted for each comparator.

**Results:**

Eleven trials (1,884 patients) were included. Remimazolam reduced respiratory depression (RR = 0.44 [95% CI 0.29; 0.67]; p = 0.0002; I² = 0%), hypoxia incidence (RR = 0.60 [95% CI 0.39; 0.93]; p = 0.0227; I² = 64.7%), bradycardia (RR = 0.39 [95% CI 0.20; 0.77]; p = 0.0069; I² = 52.3%), and hypotension (RR = 0.61 [95% CI 0.40; 0.95]; p = 0.0289; I² = 74.0%) compared to all sedatives. Compared to Propofol, Remimazolam reduced the incidence of hypotension (RR = 0.42 [95% CI 0.31; 0.58]; p < 0.0001; I² = 0%), respiratory depression (RR = 0.41 [95% CI 0.25; 0.68]; p = 0.0005; I² = 12.3%), but increased induction time (MD = 0.61 min [95% CI 0.23; 0.99]; p = 0.002; I² = 90.9%). Compared to Dexmedetomidine, it improved satisfaction (SMD = 0.23 [95% CI 0.07; 0.39]; p = 0.004; I² = 0%) and reduced recovery time (MD = -1.79 min [95% CI -2.66; -0.92]; p < 0.001; I² = 90.7%), hypoxia incidence (RR = 0.49 [95% CI 0.28; 0.88]; p = 0.0162; I² = 60.3%), and induction time (MD = -2.21 min [95% CI -2.41; -2.00]; p < 0.001; I² = 0%). Compared to Midazolam, Remimazolam increased sedation success (RR = 2.03 [95% CI 1.40; 2.95]; p = 0.0002; I² = 50%), shortened induction time (MD = -0.69 min [95% CI -1.37; -0.01]; p = 0.047; I² = 81.5%), and recovery time (MD = -4.49 min [95% CI -7.06; -1.92]; p < 0.001; I² = 40.9%).

**Conclusions:**

Remimazolam reduced respiratory depression overall and demonstrated improved safety, faster recovery, and greater efficacy compared to Propofol, Dexmedetomidine, and Midazolam, respectively, supporting its potential as an effective alternative for sedation in FB. Nonetheless, substantial heterogeneity in certain outcomes and the relatively small sample size in some comparisons limit the generalizability of our findings.

**Systematic review protocol:**

PROSPERO (CRD 42024568148).

## Introduction

Flexible Bronchoscopy (FB) is a widely performed procedure for diagnosing and treating pulmonary diseases.^1^, ^2^, ^3^ In the United States alone, over 500,000 FB procedures are done annually, underscoring the clinical importance of optimizing sedation strategies in high-volume settings.^4^ This high procedural burden is also reflected globally, where FB remains a cornerstone in the diagnosis and management of respiratory conditions, with utilization having expanded significantly worldwide over the past two decades.^5^ Ensuring optimal sedation during this procedure is critical to patient safety, comfort, and procedural success. Propofol, Dexmedetomidine and Midazolam are commonly employed to guarantee adequate sedation during FB, and Remimazolam has emerged as a promising alternative.^6^^,^^7^

Remimazolam is a novel short-acting benzodiazepine that enhances GABA_A_ receptor activity and is rapidly hydrolyzed by nonspecific esterases, forming an inactive metabolite.^8^ Propofol is an intravenous hypnotic agent that acts as a γ-aminobutyric acid-A (GABA_A_) receptor agonist and has been widely used over the past three decades.^9^ Dexmedetomidine, a selective α-2 adrenoceptor agonist approved in the United States in 1999, is commonly used for sedation.^10^ Midazolam, a benzodiazepine, is one of the most frequently used sedatives, primarily modulating GABA_A_ receptors.^11^

Due to Remimazolam’s pharmacological properties, it has emerged as an alternative for bronchoscopy sedation, highlighting its clinical relevance due to its quick onset, high procedure success rate, minimal residual effect, hemodynamic and respiratory stability.^7^^,^^12^^,^^13^ Additionally, traditional sedatives used for FB are associated with various adverse effects. Propofol is linked to hypotension, bradycardia, and respiratory depression.^6^^,^^7^^,^^14^ Dexmedetomidine may prolong recovery time.^15^ Midazolam is known to cause prolonged post-procedure sedation and delayed recovery.^6^^,^^14^ Although Remimazolam has shown promising results, there is a lack of comprehensive evidence comparing its efficacy and safety with these sedatives for FB procedure.

This systematic review and meta-analysis compares Remimazolam versus Propofol, Dexmedetomidine and Midazolam in terms of efficacy and safety outcomes ‒ hypotension, bradycardia, intraprocedural opioid consumption, hypoxia, respiratory depression, patient satisfaction, induction time, and recovery time ‒ with subgroup analyses comparing Remimazolam with each sedative individually.

## Methods

This systematic review and meta-analysis were performed and reported following the Cochrane Collaboration Handbook for Systematic Review of Interventions^16^ and the Preferred Reporting Items for Systematic Review and Meta-Analysis (PRISMA) Statement guidelines.^17^ This review was registered in the International Prospective Register of Systematic Reviews (PROSPERO) database on July 22, 2024 (registration number CRD 42024568148), https://www.crd.york.ac.uk/PROSPERO/view/CRD42024568148. There were no deviations from the pre-specified protocol in this systematic review and meta-analysis, including eligibility criteria, outcome definitions, data extraction, and statistical methods.

### Systematic literature search

We conducted a comprehensive systematic search on July 17, 2025 across PubMed, Embase, and Cochrane Library databases to identify all relevant studies. A combination of the following terms was used to search all databases: Remimazolam; Byfavo; “CNS 7056”. The complete search strategy for each database is provided in the Supplementary Appendix. Only studies in the English language were included; no further restrictions were applied to the search, and the grey literature was not searched, in order to enhance methodological rigor and ensure the reliability of the synthesized evidence.

### Selection criteria and data extraction

We included only Randomized Controlled Trials (RCTs) in English in this systematic review and meta-analysis. Two authors independently assessed the titles and abstracts using Rayyan, assessed the full texts of potentially eligible studies and removed the duplicates.^18^ We consulted a third author to reach a consensus in cases of unresolved disagreement and included studies based on the Population, Intervention, Control, and Outcomes (PICO) guidelines.^19^^,^^20^ The inclusion criteria were: 1) Population: Adult patients undergoing flexible bronchoscopy; 2) Intervention: use of intravenous Remimazolam for sedation; 3) Comparison: intravenous Propofol, Dexmedetomidine, or Midazolam for sedation; and 4) Outcomes: incidence of hypotension during the procedure, bradycardia, intraprocedural opioid consumption, respiratory depression, patient satisfaction, success of sedation, time to complete recovery of consciousness, hypoxia and induction time to sedation.

We excluded: 1) Studies with overlapping populations, defined as the same institutions and recruitment periods; 2) Studies reporting no outcomes of interest; 3) No control group; 4) Non-RCTs; 5) Patients undergoing rigid bronchoscopy; 6) Conference abstracts; and 7) Patients under conscious sedation. There were no restrictions based on the publication date. We did not search the grey literature and contacted the corresponding author for specific study results, in cases of missing data from individual studies. All included and cited studies will be systematically screened for potential retractions using the PubMed/MEDLINE and Retraction Watch databases.

Two reviewers independently collected data using a pre-designed Excel datasheet. The following variables were collected: publication year; country; enrollment period; baseline characteristics; Remimazolam dosage; comparator sedative dosages; analgesic regimen; sedation level; incidence of adverse events (hypotension, respiratory depression, bradycardia, hypoxia); intraprocedural opioid consumption; patient satisfaction, sedation success; recovery time; and induction time.

### Quality and risk assessment

Two reviewers independently evaluated the risk of bias using version 2 of the Cochrane Risk of Bias tool (RoB2) for randomized controlled trials. They assigned a judgment of “low risk”, “some concerns”, or “high risk” for each of the five domains: bias arising from the randomization process, deviations from intended interventions, missing outcome data, outcome measurement, and selection of the reported results. Any disagreements were resolved through consensus.^21^

To assess the robustness of the obtained estimates and evaluate heterogeneity, a leave-one-out analysis (Supplemental Fig. 1) was performed for all outcomes. When an outcome included at least 10 studies, we assessed publication bias through the funnel plot and Egger’s test (Supplemental Fig. 2). Additionally, Baujat plots were used to identify individual studies that contributed substantially to heterogeneity in cases where I² was 50% or higher (Supplemental Fig. 3).^22^

### Primary and secondary outcomes

Our primary outcomes were the incidence of hypotension, bradycardia and intraprocedural opioid consumption. Hypotension was defined as Systolic Blood Pressure (SBP) < 90 mmHg or 20% lower than baseline,^23^^,^^24^ or a Mean Arterial Pressure (MAP) reduction > 20% of baseline.^23^, ^24^, ^25^, ^26^ Bradycardia was defined as a Heart Rate (HR) < 60 Beats Per Minute (BPM) or a reduction greater than 20% compared with baseline or an HR < 50 BPM.^25^^,^^26^ Intraprocedural opioid consumption was expressed in intravenous morphine equivalents, calculated using the method proposed by Kane et al., following the guidelines of the American Pain Society.^27^

We pooled results whenever three or more studies reported the same outcome. The secondary outcomes included respiratory depression, defined as < 10 breaths per minute or < 8 breaths per minute;^25^ hypoxia, defined as oxygen saturation < 90%.^23^, ^24^, ^25^, ^26^ Patient satisfaction score was assessed by a standardized 5-point or 10-point scale, where lower scores indicate lower satisfaction; induction time; sedation success, defined as successful completion of the FB procedure; and time to complete recovery of consciousness, defined as the interval from the bronchoscopy completion to full awakening.

### Statistical analysis

We analyzed the data using R software, version 4.4.0. We computed Risk Ratios (RR) with 95% Confidence Intervals (95% CI) for each binary endpoint. We defined statistical significance as p < 0.05. We reported continuous outcomes as Mean Differences (MD) with 95% CI when studies used the same scale and as Standardized Mean Differences (SMD) with 95% CI when different scales were used to measure the same outcome. When continuous data were reported as medians with IQR or range, we calculated the mean and SD using the methods proposed by Luo et al. and Wan et al.^28^^,^^29^ Considering the anticipated clinical and methodological heterogeneity across studies, including variability in patient populations, dosing regimens, and comparators, a random-effects model was deemed the most suitable approach for synthesizing the pooled results.^28^^,^^29^ Additionally, we assessed heterogeneity among the trials using the I² statistic, with I² > 50% indicating significant heterogeneity and explored heterogeneity sources with leave-one-out sensitive analysis and Baujat plot.^22^^,^^30^ We conducted subgroup analyses for each comparator (Propofol, Dexmedetomidine and Midazolam) to distinguish their individual effects on the pooled results. The data and analytical code supporting the findings of this study are available from the corresponding author upon reasonable request.

## Results

### Search results

**Figure 1:**
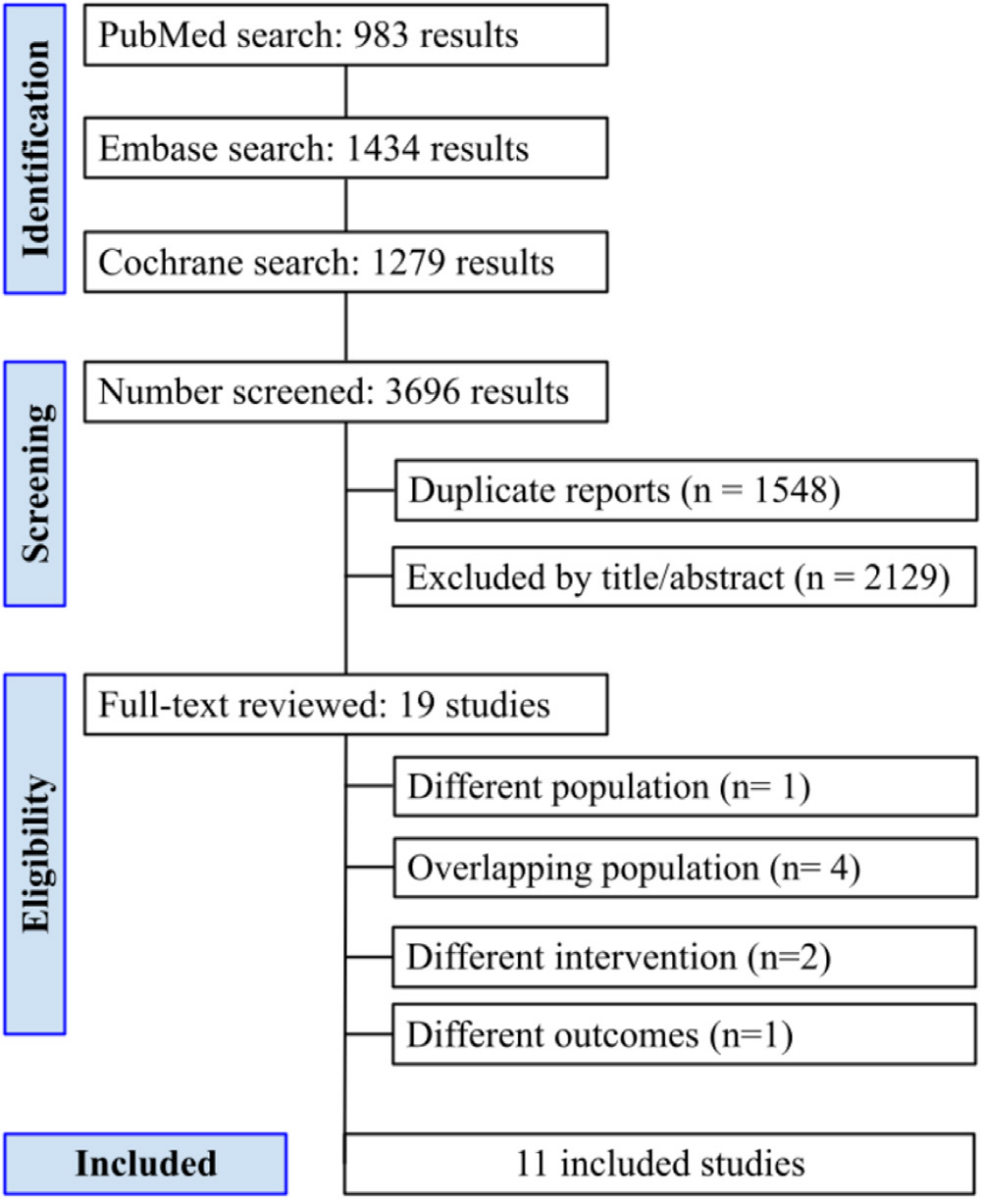
PRISMA-compliant flow diagram of study screening and selection. PRISMA, Preferred Reporting Items for Systematic Reviews and Meta-analysis.

We identified a total of 3,696 reports through our search strategy, with 1,548 duplicates. We screened the titles and abstracts of the remaining 2,148 reports, excluding 2,129. We assessed the full text of nineteen trials, excluding eight (Fig. 1). The remaining eleven RCTs were included in this systematic review and meta-analysis.

### Study characteristics

We included eleven RCTs with 1,884 patients.^23^, ^24^, ^25^, ^26^^,^^31^, ^32^, ^33^, ^34^, ^35^, ^36^, ^37^ Of these, 1,057 received Remimazolam, while 827 received comparator sedatives, including Dexmedetomidine, Propofol, and Midazolam. All patients were classified as ASA I‒III. Nine studies were conducted in China. The dose of Remimazolam varied across the RCTs. Details of the individual studies, like drug dosages and relevant characteristics, are summarized in Table 1.

**Table 1:** Characteristics of the included studies.

Study (Author, Year)	Country	Enrollment Period	ASA	Sample Size (Remimazolam/Control)	Age (years), mean ± SD	Body Weight (kg), mean ± SD	Remimazolam Details	Control Details	Analgesia Protocol	Procedure Details	Benzodiazepine antagonists	Outcomes assessed	Level of Sedation
Chen, 2022	China	December 2020 to November 2021	I‒II	73/73	56.55 ± 5.80	Remimazolam group: 68.97 ± 5.33. Dexmedetomidine group: 69.64 ± 6.20	First 10 min: 2000 μg.kg^-1^ Maintenance: 16.7–33.3 μg.kg^-1^.min^-1^	First 10 min: Dexmedetomidine 0.5 μg.kg^-1^ Maintenance: Dexmedetomidine 0.003–0.012 μg.kg^-1^.min^-1^	Single dose: Fentanyl 1 μg.kg^-1^, Dexamethasone 0.1 mg.kg^-1^ Infusion: Remifentanil 0.05–0.2 μg.kg^-1^.min^-1^	Endobronchial inspection, bronchoscopic biopsy and lavage	All patients: flumazenil (200 μg)	Sedation success, respiratory depression, time to complete recovery of consciousness, patient satisfaction, hypoxia, hypotension, bradycardia	Moderate
Xu, 2024	China	April to September 2023	I‒II	60/60	60.80 ± 4.23	Remimazolam group: 67.2 ± 6.2 Dexmedetomidine group: 67.6 ± 7.4	First 10 min: 1000 μg.kg^-1^ Maintenance: 16.7–33.3 μg.kg^-1^.min^-1^	First 10 min: Dexmedetomidine 0.5 μg.kg^-1^ Maintenance: Dexmedetomidine 0.003–0.012 μg.kg^-1^.min^-1^	5 min before induction: Alfentanil 10 μg.kg^-1^ During procedure: Alfentanil 0.5–2 μg.kg^-1^.min^-1^	Endobronchial inspection, bronchoscopic biopsy and lavage	N/A	Sedation success, time to complete recovery of consciousness, patient satisfaction, intraprocedural opioid consumption, induction time, hypoxia, hypotension, bradycardia	Moderate
Gao, 2023	China	January 2021 to August 2021	I‒III	30/30	59.4 ± 9.15	N/A	Induction: 100 μg.kg^-1^.min^-1^ Maintenance: 10–33.3 μg.kg^-1^.min^-1^ Rescue: 100 μg.kg^-1^	Induction: Propofol 2000 μg.kg^-1^ Maintenance: Propofol 66.7–100 μg.kg^-1^.min^-1^ Rescue: 500 μg.kg^-1^	5 min prior: Sufentanil 0.15 μg.kg^-1^	Elective bronchoscopy	N/A	Time to complete recovery of consciousness, patient satisfaction, induction time, hypoxia, hypotension, bradycardia	Deep
Zhang, 2023	China	N/A	I‒III	96/96	64.37 ± 13.38	Remimazolam group: 56.72 ± 12.37 Propofol group: 58.72 ± 11.31	Single dose: 200 μg.kg^-1^ Rescue: Remimazolam 50 μg.kg^-1^	Single dose: Propofol 1500 μg.kg^-1^ Rescue: Propofol 500-1000 μg.kg^-1^	Prior anesthesia: Alfentanil 10 μg.kg^-1^	Bronchoscopy with preserved spontaneous breathing	N/A	Respiratory depression, time to complete recovery of consciousness, patient satisfaction, intraprocedural opioid consumption, induction time, hypotension, bradycardia	Moderate
Zhou, 2022	China	N/A	I‒III	155/155	50.76 ± 12.65	Remimazolam group: 60.8 ± 9.57 Propofol group: 62.0 ± 9.71	Single dose: 200 μg.kg^-1^ Rescue: 100 μg.kg^-1^	Initial dose: Propofol 2000 μg.kg^-1^ Rescue: Propofol 750 μg.kg^-1^	Single dose: Fentanyl 2 μg.kg^-1^	Check the airways, biopsy, EBUS-TBNA, bronchoalveolar lavage, bronchial foreign body removal	N/A	Sedation success, respiratory depression, time to complete recovery of consciousness, intraprocedural opioid consumption, hypoxia, hypotension,	Moderate
Wu, 2024	China	May 2022 to July 2022	I‒III	46/48	69.78 ± 3.86	Remimazolam group: 59.33 ± 11.91 Midazolam group: 60.67 ± 10.53	Single dose: 135 μg.kg^-1^ Rescue: titrated Propofol, until RSS = 4	Single dose: Midazolam 45 μg.kg^-1^ Rescue: titrated Propofol, until RSS = 4	Single dose: Alfentanil 1.8 μg.kg^-1^	Diagnostic flexible bronchoscopy	Flumazenil (05 μg.kg^-1^) and naloxone (3 μg.kg^-1^)	Sedation success, intraprocedural opioid consumption, induction time, hypoxia, hypotension	Deep
Kim, 2023	South Korea	April 2022 to 2023	I‒III	49/51	66.29 ± 13.53	N/A	< 60 years or > 50 kg, single dose: 5000 μg ≥ 60 years or f < 50 kg, single dose: 3000 μg Rescue: Remimazolam 2500 μg	< 60 years or > 50 kg, single dose: Midazolam 3000 μg ≥ 60 years or < 50 kg, single dose: Midazolam 2000 μg Rescue: Midazolam 500 μg	N/A	Diagnostic or therapeutic flexible bronchoscopy	N/A	Time to complete recovery of consciousness, patient satisfaction, induction time, hypoxia, hypotension, bradycardia	Moderate
Pastis, 2019	USA	N/A	I‒III	303/69	62.32 ± 12.46	Remimazolam group: 80.9 ± 20.21 Midazolam group: 83.0 ± 22.10	Initial dose: 5000 μg Top-up dose: 2500 μg	Initial dose: < 60 years Midazolam 1750 μg. > 60 years or debilitated Midazolam 1000 μg top-up dose: < 60 years Midazolam 1000 μg; > 60 years or debilitated Midazolam 500 μg	Initial dose: Fentanyl 25–75 μg Top-up dose: Fentanyl 25–200 μg	Diagnostic or therapeutic flexible bronchoscopy	N/A	Sedation success, respiratory depression, time to complete recovery of consciousness, intraprocedural opioid consumption, hypoxia, hypotension, bradycardia	Moderate
Chai, 2025	China	March 2023 to April 2024	I‒II	30/30	69.95 ± 4.22	Remimazolam group: 60.2 ± 10.0 Propofol group: 61.8 ± 8.9	Initial dose: 200 μg.kg^-1^ Maintenance: 16.7 μg.kg^-1^.min^-1^	Initial dose: 2000 μg.kg^-1^ of propofol Maintenance: 66.7 μg.kg^-1^.min^-1^ of propofol.	0.15 μg.kg^-1^ sufentanil over 30 seconds	Fibreoptic bronchoscopy	N/A	Sedation success, respiratory depression, time to complete recovery of consciousness, patient satisfaction, hypotension, bradycardia	Deep
Luo, 2025	China	April 2024 to June 2024.	II‒III	33/33	71.4 ± 3.83	Remimazolam group: 68.6 ± 6.8 Propofol group: 67.9 ± 7.8	Initial dose: 200 μg.kg^-1^ Additional dose: 50 μg.kg^-1^	Initial dose: 1500 μg.kg^-1^ of propofol Additional dose: 500 μg.kg^-1^ of propofol	10 μg.kg^-1^ of alfentanil over 30 seconds	Flexible fibreoptic bronchoscopy	N/A	Sedation success, time to complete recovery of consciousness, hypoxia, hypotension, bradycardia	Deep
Zhou, 2024	China	April 2021 to September 2022.	I‒III	182/182	59.8 ± 9	Remimazolam group: 61.7 ± 11.9 Dexmedetomidine group: 60.3 ± 11.1	Initial dose: 100‒200 μg.kg^-1^ Maintenance dose: to 1.7–8.3 μg.kg^-1^.min^-1^	Initial dose: 0.4–0.8 μg.kg^-1^ of dexmedetomidine Maintenance dose: 0.0067–0.0333 μg.kg^-1^.min^-1^ of dexmedetomidine	0.1 μg.kg^-1^.min^-1^ of remifentanil and ideally shouldn’t be > 0.2 μg.kg^-1^.min^-1^	Flexible fibreoptic bronchoscopy	N/A	Time to complete recovery of consciousness, patient satisfaction, intraprocedural opioid consumption, induction time, hypoxia, hypotension, bradycardia	Deep

### Risk of bias

**Figure 2:**
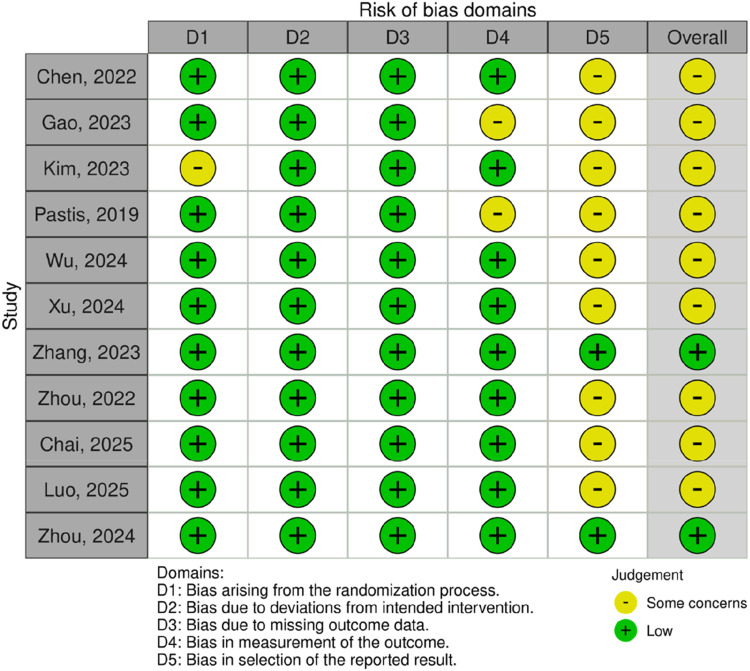
Risk of bias assessment with Cochrane’s risk of bias tool for randomized trials.

Among the eleven included studies in this systematic review and meta-analysis, two were assessed as having an overall low risk of bias, while the remaining nine were classified as presenting “some concerns”, predominantly due to limitations in the domain of selective reporting of results. Importantly, no study was deemed to have a high overall risk of bias. A summary of the assessment for each specific domain is provided in Figure 2.

The primary source of bias concerns was incomplete or unclear reporting of prespecified outcomes, which may introduce a risk of reporting bias. Nevertheless, all studies provided sufficient data for the extraction and analysis of primary and secondary outcomes. Moreover, domains critical to internal validity, including the randomization process, adherence to intended interventions, completeness of outcome data, and outcome measurement, were predominantly judged to be at low risk, thereby enhancing the overall reliability of the evidence base.

### Outcomes

#### Primary outcomes

**Figure 3:**
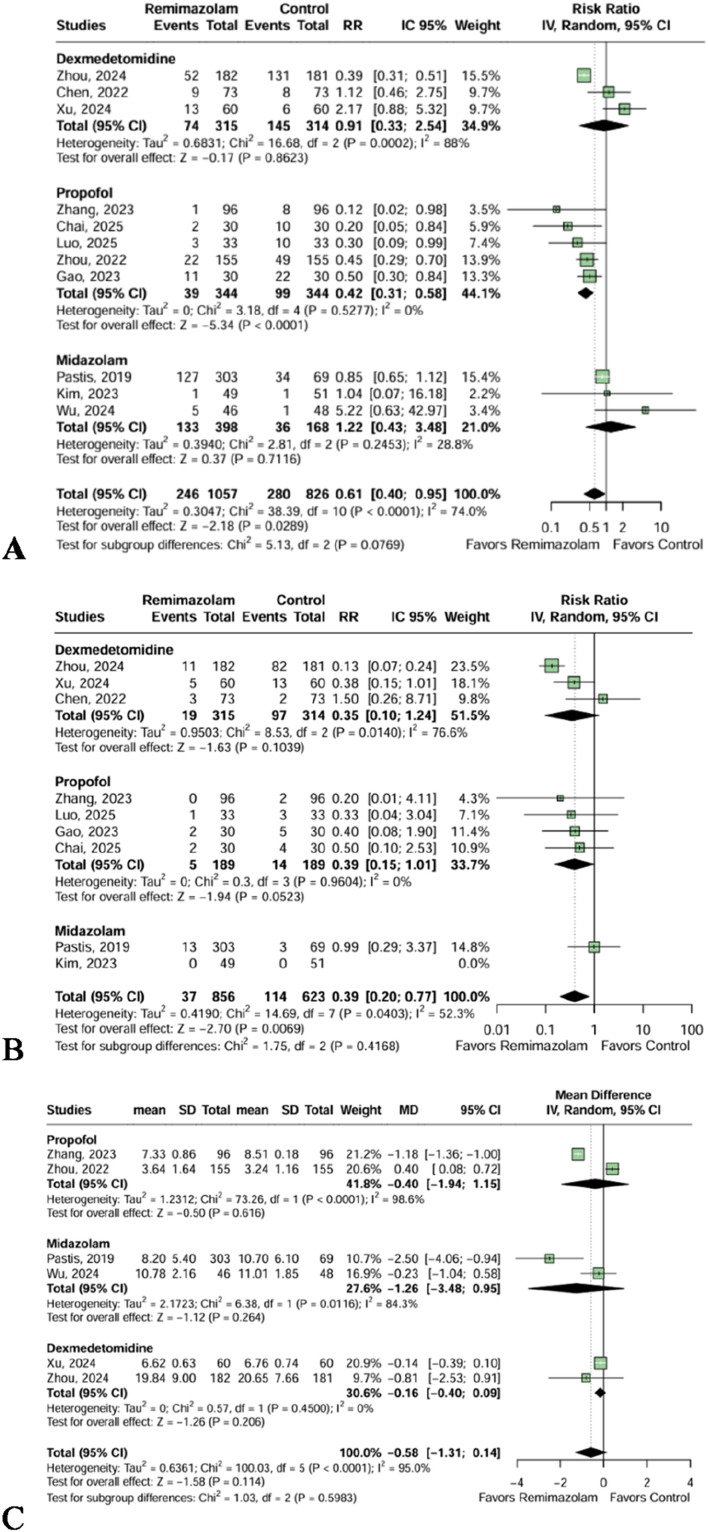
(A) The incidence of hypotension was significantly reduced in the Remimazolam group compared to Propofol and in the overall analysis. (B) The Remimazolam group showed a significant reduction in bradycardia in the overall analysis. (C) Intraprocedural opioid consumption, in milligrams of intravenous morphine, did not present a statistical difference between Remimazolam and studied groups.

The Remimazolam group demonstrated a statistically significant reduction in the incidence of hypotension (RR = 0.61 [95% CI 0.40; 0.95]; p = 0.0289, I² = 74.0%, 11 trials, 1,883 patients, Fig. 3A). When individually compared to Propofol, the Remimazolam group was associated with a significantly lower incidence of hypotension (RR = 0.42 [95% CI 0.31; 0.58]; p < 0.0001, I² = 0%, 5 trials, 688 patients, Fig. 3A). No significant differences were observed between Remimazolam and Dexmedetomidine or Midazolam in the subgroup analysis for this outcome. The certainty of evidence was deemed low for the hypotension incidence outcome.

Remimazolam showed a statistically significant reduction in the incidence of bradycardia in the overall analysis (RR = 0.39 [95% CI 0.20; 0.77]; p = 0.0069, I² = 52.3%, 9 trials, 1479 patients, Fig. 3B). The certainty of evidence was deemed low for the bradycardia incidence outcome. The subgroup analysis showed no significant difference between Remimazolam and any individual comparator for this outcome.

Similarly, intraprocedural opioid consumption did not significantly differ between Remimazolam and the overall sedative group (MD = -0.58 mg of intravenous morphine [95% CI -1.31; 0.14]; p = 0.114, I² = 95%, 6 trials, 1,451 patients, Fig. 3C), with no statistical differences found in the subgroup comparisons. The certainty of evidence was deemed low for the intraprocedural opioid consumption outcome.

#### Secondary outcomes

**Figure 4:**
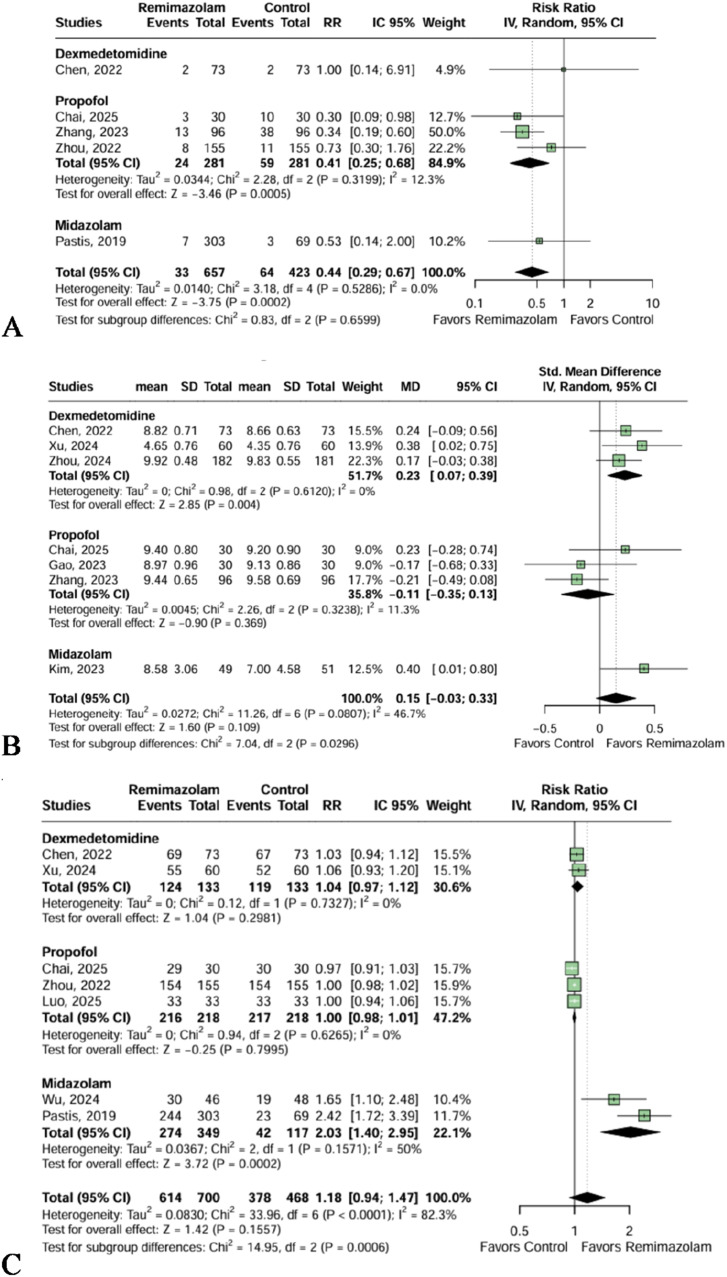
(A) Respiratory depression was statistically decreased in the Remimazolam group compared to the overall analysis and compared to Propofol. (B) The Remimazolam group demonstrated a significantly higher patient satisfaction score, in comparison to Dexmedetomidine. (C) The Remimazolam group presented a statistically higher success of sedation compared to Midazolam.

The Remimazolam group showed a statistical reduction in respiratory depression compared to the overall analysis (RR = 0.44 [95% CI 0.29; 0.67]; p = 0.0002, I² = 0%, 5 trials, 1080 patients, Fig. 4A) and when individually compared to Propofol (RR = 0.41 [95% CI 0.25; 0.68]; p = 0.0005, I² = 12.3%, 3 trials, 562 patients, Fig. 4A). No significant differences were observed with the other sedatives. The certainty of evidence was deemed moderate for the respiratory depression incidence outcome.

Our analysis revealed no statistically significant difference between the Remimazolam group and the overall analysis on the patient satisfaction score (SMD = 0.15 [95% CI -0.03; 0.33]; p = 0.109, I² = 46.7%, 7 trials, 1041 patients, Fig. 4B). However, Remimazolam sedation was associated with higher patient satisfaction compared to the Dexmedetomidine individual group (SMD = 0.23 [95% CI 0.07; 0.39]; p = 0.004, I² = 0%, 3 trials, 629 patients, Fig. 4B). There was no significant difference between Remimazolam and the other comparators. The certainty of evidence was deemed moderate for the patient satisfaction score outcome.

No significant difference was observed for sedation success between Remimazolam and the overall analysis (RR = 1.18 [95% CI 0.94; 1.47; p = 0.1557, I² = 82.3%, 7 trials, 1168 patients, Fig. 4C). However, subgroup analysis showed a significantly higher success rate with Remimazolam compared to Midazolam (RR = 2.03 [95% CI 1.40; 2.95]; p = 0.0002, I² = 50%, 2 trials, 466 patients, Fig. 4C), but no significant difference with Propofol or Dexmedetomidine. The certainty of evidence was deemed low for sedation success outcome.

**Figure 5:**
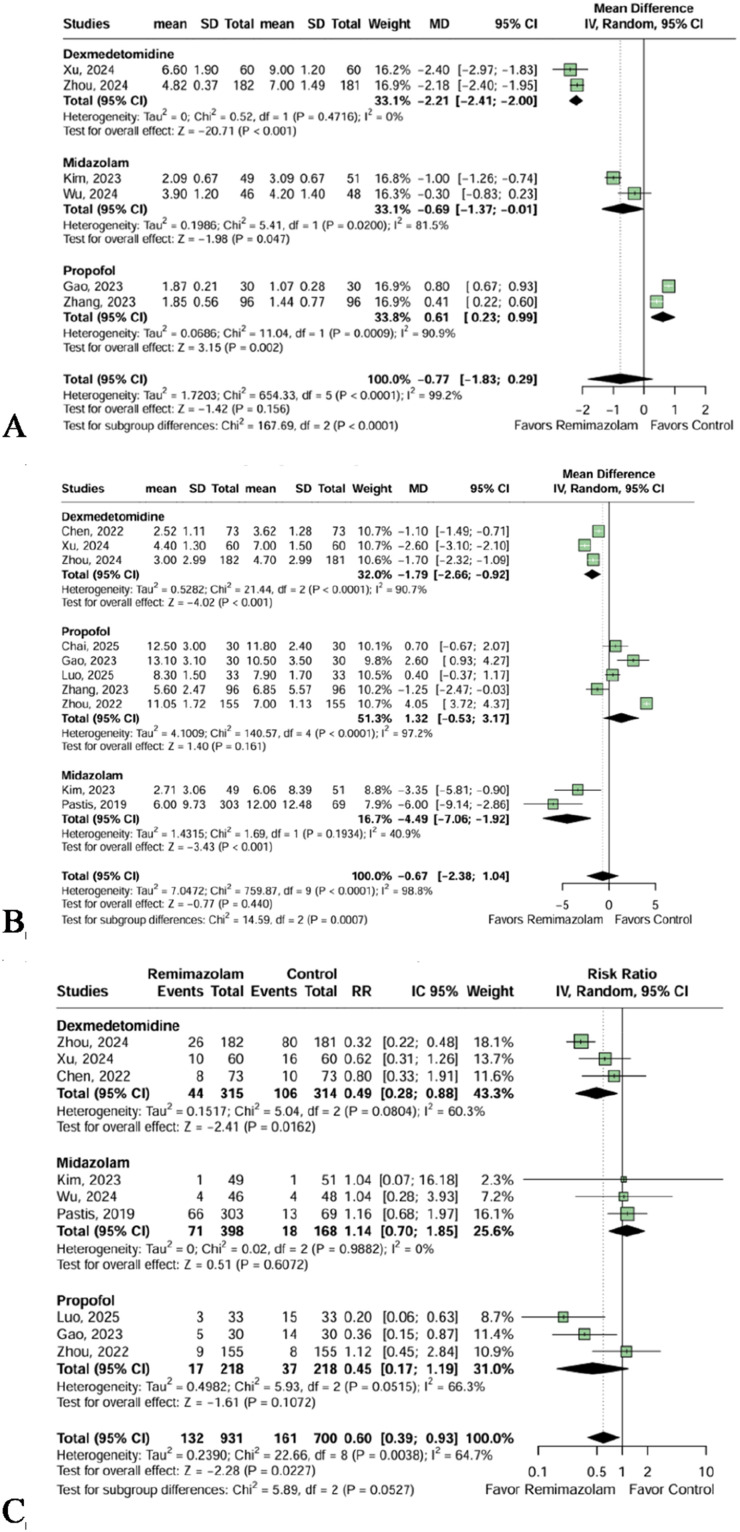
(A) Induction time, in minutes, was significantly reduced in the Remimazolam group compared to Midazolam and Dexmedetomidine. Remimazolam sedation presented a statistically higher induction time compared with Propofol. (B) Time to complete recovery of consciousness, in minutes, was statistically faster in the Remimazolam group, in comparison to Midazolam and Dexmedetomidine. (C) Hypoxia was significantly reduced in the Remimazolam group compared to Dexmedetomidine and in the overall analysis.

Overall, there was no difference in induction time (MD = -0.77 minutes [95% CI -1.83; 0.29]; p = 0.156, I² = 99.2%, 6 trials, 929 patients, Fig. 5A), although our subgroup analysis demonstrated Remimazolam had a significant shorter induction time compared to Midazolam (MD = -0.69 minutes [95% CI -1.37; -0.01]; p = 0.047, I² = 81.5%, 2 trials, 194 patients, Fig. 5A) and Dexmedetomidine (MD = -2.21 minutes [95% CI -2.41; -2.00]; p < 0.001, I² = 0%, 2 trials, 483 patients, Fig. 5A). The comparison with Propofol demonstrated a longer induction time (MD = 0.61 minutes [95% CI 0.23; 0.99] p = 0.002, I² = 90.9%, 2 trials, 252 patients, Fig. 5A). The certainty of evidence was deemed low for induction time outcome.

No significant difference was observed between the Remimazolam and the pooled comparator group in the time to complete recovery of consciousness (MD = -0.67 minutes [95% CI -2.38; 1.04], p = 0.440, I² = 98.8%, 10 trials, 1789 patients, Fig. 5B). However, we noted a significant faster recovery with Remimazolam compared to Midazolam (MD = -4.49 minutes [95% CI -7.06; -1.92], p < 0.001, I² = 40.9%, 2 trials, 472 patients, Fig. 5B) and Dexmedetomidine (MD = -1.79 minutes [95% CI -2.66; -0.92], p < 0.001, I² = 90.7%, 3 trials, 629 patients, Fig. 5B). In contrast, no difference was found with Propofol for this outcome (MD = 1.32 minutes [95% CI -0.53; 3.17] p = 0.161, I² = 97.2%, 5 trials, 688 patients, Fig. 5B). The certainty of evidence was deemed low for the time to complete recovery of consciousness outcome.

Furthermore, Remimazolam's incidence of hypoxia was statistically lower when compared with the overall sedatives (RR = 0.60 [95% CI 0.39; 0.93]; p = 0.0227, I² = 64.7%, 9 trials, 1631 patients, Fig. 5C). When compared specifically with dexmedetomidine, Remimazolam also presented a significant reduction in hypoxia events (RR = 0.49 [95% CI 0.28; 0.88]; p = 0.0162, I² = 60.3%, 3 trials, 629 patients, Fig. 5C). No significant difference was found in the comparison of Remimazolam and Midazolam or Propofol. The certainty of evidence was deemed low for the hypoxia outcome.

### Sensitivity analysis

The robustness of the finding regarding hypotension was further evaluated through a leave-one-out sensitivity analysis and a Baujat plot. The pooled RRs ranged from 0.54 to 0.67 (95% CI 0.35 to 1.09). However, statistical significance was not maintained in most analyses, with a loss of significance observed upon exclusion of Zhou et al. (2024), Gao et al. (2023), Zhang et al. (2023), Zhou et al. (2022), Chai et al. (2025), and Luo et al. (2025). Heterogeneity remained relatively stable across exclusions, ranging from 64.8% to 76.4. (Supplemental Fig. 1A) The Baujat analysis revealed Zhou et al. (2024) and Pastis et al. (2019) as the studies with the largest contribution to both heterogeneity and influence on overall result (Supplemental Fig. 2A).

The bradycardia overall pooled analysis demonstrated consistent results across the leave-one-out sensitivity analysis, with RR ranging from 0.33 to 0.54 (95% CIs between 0.16 and 0.95). Notably, the exclusion of Zhou et al. (2024) resulted in a substantial reduction in heterogeneity (I² = 0%). However, the omission of any single study did not alter the direction or significance of the pooled effect, reinforcing the robustness of the findings (Supplemental Fig. 1B). The Baujat analysis reinforced Zhou et al. (2024) as the study with the highest contribution to both heterogeneity and influence on the overall result (Supplemental Fig. 2B).

Intraprocedural opioid consumption heterogeneity was not statistically influenced by any single study, with MD ranging from -0.81 to -0.35 milligrams of intravenous morphine (95% CIs between -1.64 and 0.39). Statistical significance favoring Remimazolam was observed only when Zhou et al. (2022) was excluded (MD = -0.81, [95% CI -1.52; -0.10], I² = 92.3%). Overall heterogeneity remained high throughout the analysis (I² = 78.3% to 96.0%), indicating that no single study disproportionately influenced between-study variability (Supplemental Fig. 1C). However, the Baujat plot revealed Zhang et al. (2023) as the study with the largest contribution to both heterogeneity and influence on the overall result (Supplemental Fig. 2C).

The respiratory depression overall pooled analysis had low heterogeneity (I² = 0%), with individual studies' RR ranging from 0.38 to 0.56 (95% CIs between 0.24 and 1.02). Leave-one-out showed that omitting Zhang et al. (2023) led to the loss of statistical significance (RR = 0.56 [95% CI 0.31; 1.02], I² = 0%), indicating its substantial contribution to the observed effect. In contrast, removing Pastis et al. (2019) slightly increased heterogeneity (I² = 2.5%), but did not affect statistical significance (Supplemental Fig. 1D). As the heterogeneity for this outcome was considerately low, a Baujat plot was not generated in accordance with our predefined methodological strategy.

Patient satisfaction score leave-one-out analysis revealed that removing Zhang et al. (2023) reduced heterogeneity substantially from 46.7% to 0% and led to statistical significance favoring Remimazolam (SMD = 0.22 [95% CI 0.09; 0.36], I² = 0%). Overall SMD ranged from 0.11 to 0.22 (95% CIs between -0.09 and 0.37) (Supplemental Fig. 1E). Since the heterogeneity for this outcome was below 50 percent, a Baujat plot was not assessed, consistent with our predefined methodological strategy.

Hypoxia had a RR range from 0.52 to 0.70 (95% CIs between 0.34 and 1.07). Loss of statistical significance occurred when excluding Zhou et al. (2024), Luo et al. (2025), or Gao et al. (2023), indicating these studies contributed meaningfully to the overall significance. Heterogeneity remained relatively stable across exclusions, with I² values ranging from 40.0% to 68.9% (Supplemental Fig. 1F). The Baujat plot revealed Zhou et al. (2024) as the study with the largest contribution to both heterogeneity and influence on the overall result (Supplemental Fig. 2D).

For induction time, high heterogeneity persisted regardless of which studies were excluded. Notably, exclusion of Gao et al. (2023) resulted in a statistically significant reduction in induction time favoring the Remimazolam group (MD = -1.09 [95% CI -2.14; -0.03] I² = 98.8%) (Supplemental Fig. 1G). The Baujat plot revealed Gao et al. (2023) as the study exerting the greatest influence on the pooled effect, whereas Zhou et al. (2024) accounted for the largest contribution to heterogeneity (Supplemental Fig. 2E).

Leave-one-out sensitivity analysis for sedation success revealed no significant changes in either heterogeneity or effect estimates, with RR range from 1 to 1.22 (95% CIs between 0.90 and 1.60) (Supplemental Fig. 1H). The Baujat plot revealed Zhou et al. (2022) as the study exerting the greatest influence on the pooled effect, whereas Pastis et al. (2019) accounted for the largest contribution to heterogeneity (Supplemental Fig. 2F).

For time to complete recovery of consciousness, high heterogeneity persisted regardless of which studies were excluded, with no impact on the direction or significance of the combined effect, with MD range from -1.18 to -0.21 (95% CIs between -2.77 and 1.40) (Supplemental Fig. 1I). The Baujat plot revealed Zhou et al. (2022) as the study with the largest contribution to both heterogeneity and influence on the overall result (Supplemental Fig. 2G).

Funnel plot analysis and Egger’s test were performed for the outcomes of hypotension and time to complete recovery of consciousness, as both included at least 10 studies. The funnel plots demonstrated symmetrical distributions around the pooled effect estimates, and Egger’s test did not indicate evidence of publication bias for either outcome (Supplemental Fig. 3).

### Certainty of evidence

According to the GRADE approach, the initial certainty of the evidence was considered high, as all included studies were randomized controlled trials. The certainty was subsequently downgraded based on the assessment of risk of bias, inconsistency, imprecision, and potential publication bias. Respiratory depression incidence, and patient’s satisfaction score were judged to have moderate certainty, whereas hypotension, bradycardia, intraprocedural opioid consumption, success of sedation, induction time, time to complete recovery of consciousness, and hypoxia were classified as low certainty. A detailed GRADE assessment and summary of findings are presented in Table 2.

**Table 2:** GRADE assessment and summary of findings. Author(s): Question: Remimazolam compared to Dexmedetomidine, Midazolam and Propofol for bronchoscpy Setting: Bibliography.

Certainty assessment							N° of patients		Effect		Certainty	Importance
N° of studies	Study design	Risk of bias	Inconsistency	Indirectness	Imprecision	Other considerations	[Intervention]	[Comparison]	Relative (95% CI)	Absolute (95% CI)		
**Hypotension**												
11	Randomized trials	Serious^a^	Serious	Not serious	Not serious	None	246/1057 (23.3%)	280/826 (33.9%)	**RR 0.61** (0.40 to 0.95)	132 fewer per 1.000 (from 203 fewer to 17 fewer)	⨁⨁○○ Low^a^	Important
**Bradycardia**												
9	Randomized trials	Serious^a^	Serious^b^	Not serious	Not serious	None	37/856 (4.3%)	114/623 (18.3%)	**RR 0.39** (0.20 to 0.77)	112 fewer per 1.000 (from 146 fewer to 42 fewer)	⨁⨁○○ Low^a^^,^^b^	Important
**Intraprocedural opioid consumption**												
6	Randomized trials	Serious^a^	Serious^b^	Not serious	Not serious	None	842	609	‒	MD **0.58 mg of IV morphine equivalent lower** (1.31 lower to 0.14 higher)	⨁⨁○○ Low^a^^,^^b^	Important
**Respiratory depression**												
5	Randomized trials	Serious^a^	Not serious	Not serious	Not serious	None	33/657 (5.0%)	64/423 (15.1%)	**RR 0.44** (0.29 to 0.67)	85 fewer per 1.000 (from 107 fewer to 50 fewer)	⨁⨁⨁○ Moderate^a^	Important
**Patient satisfaction score**												
7	Randomized trials	Serious^a^	Not serious	Not serious	Not serious	None	520	521	‒	SMD **0.15 SD higher** (0.03 lower to 0.33 higher)	⨁⨁⨁○ Moderate^a^	Important
**Success of sedation**												
7	Randomized trials	Serious^a^	Serious^b^	Not serious	Not serious	None	614/700 (87.7%)	378/468 (80.8%)	**RR 1.10** (0.04 to 1.47)	81 more per 1.000 (from 775 fewer to 380 more)	⨁⨁○○ Low^a^^,^^b^	Important
**Induction time**												
6	Randomized trials	Serious^a^	Serious^b^	Not serious	Not serious	None	463	466	‒	MD **0.77 minutes lower** (1.83 lower to 0.29 higher)	⨁⨁○○ Low^a^^,^^b^	Important
**Time to complete recovery of consciouness**												
10	Randomized trials	Serious^a^	Serious^b^	Not serious	Not serious	None	1011	778	‒	MD **0.67 minutes lower** (2.38 lower to 1.04 higher)	⨁⨁○○ Low^a^^,^^b^	Important
**Hypoxia**												
9	Randomized trials	Serious^a^	Serious^b^	Not serious	Not serious	None	132/931 (14.2%)	161/700 (23.0%)	**RR 0.60** (0.39 to 0.93)	92 fewer per 1.000 (from 140 fewer to 16 fewer)	⨁⨁○○ Low^a^^,^^b^	Important

CI, Confidence Interval; MD, Mean Difference; RR, Risk Ratio; SMD, Standardized Mean Difference.

Explanations:

a Outcome significantly carried out by studies with “some concerns” risk of bias. Downgraded by one level.

b High heterogeneity (I^2^ > 50%). Downgraded by one level for inconsistency.

## Discussion

In this systematic review and meta-analysis, including 1,884 patients from 11 RCTs, we found that Remimazolam was associated with a significantly lower risk of respiratory depression, hypoxia, hypotension, and bradycardia compared to commonly used sedatives. Subgroup analyses showed that 1) Compared to Propofol, Remimazolam reduced the incidence of hypotension and respiratory depression, and increased induction time; 2) Compared to Dexmedetomidine, it resulted in higher patient satisfaction, faster recovery of consciousness, and induction time, as well as a statistically lower hypoxia incidence; and 3) Compared to Midazolam, it achieved higher sedation success, shorter induction time, and faster recovery. No significant differences were observed between groups regarding bradycardia, or intravenous morphine consumption.

The reduced incidence of hypotension and bradycardia associated with Remimazolam, compared to the general sedative group and reduced hypotension compared to Propofol, can be attributed to its selective action on GABA_A_ receptors while maintaining a negligible effect on sympathetic tone.^38^^,^^39^ In contrast, Propofol induces a dose-dependent decrease in blood pressure through the suppression of sympathetic activity, resulting in vasodilation and a reduction in peripheral vascular resistance.^40^ Furthermore, Remimazolam experiences rapid biotransformation by tissue esterases into a pharmacologically inactive metabolite (CNS 7054) and is characterized by a brief context-sensitive half-life, which reinforces its swift recovery profile in contrast to Midazolam and Dexmedetomidine.^38^ The reduction in respiratory depression in the Remimazolam group compared to both the overall, and Propofol groups can be explained by its higher respiratory depression threshold compared to Propofol.^12^

The advantageous pharmacokinetic attributes of Remimazolam likely enhance patient satisfaction, promote a quicker induction, and facilitate a more rapid recovery when compared with Dexmedetomidine, alongside superior sedation outcomes and reduced induction and recovery times when contrasted with Midazolam. Overall, the differences in pharmacodynamics and pharmacokinetics provide insight into the enhanced safety and efficacy of Remimazolam, as indicated in this meta-analysis, especially in clinical contexts where cardiorespiratory stability and procedural efficiency are critical.

Our meta-analysis provides a more comprehensive and robust evaluation of Remimazolam compared to the work by Zhou et al. Our broad search strategy identified 3,696 initial reports, contrasting their 40 initial reports, allowing us to include more than double the number of trials: 11 RCTs comprising 1,884 patients, compared to their 5 RCTs with 1,080 patients. Methodologically, our analysis incorporated a leave-one-out sensitivity analysis and Baujat plots to test the stability of our findings, a step not reported by Zhou et al. Furthermore, we assessed a wider array of clinically relevant outcomes, including induction time, recovery time, and patient satisfaction, which were not addressed in their study. While our findings were largely concordant, with both analyses concluding that Remimazolam is associated with a reduced incidence of hypotension and respiratory depression, as well as a higher success rate than Midazolam, our more powerful analysis also detected a significant reduction in bradycardia, a finding not observed by Zhou et al. Notably, while their analysis suggested a reduction in hypoxia compared to Propofol, our larger analysis did not find this effect to be statistically significant, indicating that our conclusion is likely the more reliable estimate.^37^

Our findings indicate that Remimazolam may serve as an effective, reliable, and safe sedative for flexible bronchoscopy, providing a clearer understanding of the potential trade-offs associated with selecting a sedative. For instance, Propofol acts rapidly; however, it may increase the likelihood of hypotension and respiratory complications. Conversely, Remimazolam appears to exhibit a superior safety profile, evidenced by a substantial reduction in serious events during the procedure, including a 40% decrease in hypotension and hypoxia, as well as a diminished incidence of bradycardia. This safer profile may be particularly beneficial for elderly individuals or those with additional health concerns.

Compared to Midazolam, Remimazolam demonstrated higher success of sedation, faster induction, and shorter recovery times by approximately 4.5 minutes. In clinical practice, where an average of ten FB procedures is performed daily without transbronchial needle aspiration, each session lasts around 19 minutes and costs USD 289.00.^41^^,^^42^ Our analysis reveals a total inefficiency of 5.18 minutes per procedure, resulting from a 0.69-minute longer induction and 4.49 minutes of delayed recovery compared to Midazolam. This results in a daily cost impact of roughly USD 788.71. We suggest that Remimazolam could serve as a useful alternative, enhancing patient safety and workflow efficiency. Future large-scale RCTs should establish standardized dosing protocols and evaluate performance in vulnerable populations, such as patients with severe cardiopulmonary disease and morbid obesity. Pharmacoeconomic analyses are paramount for determining whether benefits translate into cost savings, and research on long-term cognitive effects is essential for clarifying its role in procedural sedation.

A major limitation was the high heterogeneity in some outcomes, including hypotension and hypoxia, which our sensitivity analysis and Baujat plot revealed was primarily driven by two methodologically distinct trials: Pastis et al. and Zhou et al. (2024) (Supplemental Fig. 2).^32^^,^^37^ These studies diverge significantly from the other nine trials in our analysis. Pastis et al. stands out as the only study conducted in a US population, using a bolus-only dosing strategy and Midazolam as the comparator, with a primary endpoint of procedural success. Conversely, Zhou et al. (2024) is unique for its use of Dexmedetomidine as the comparator and the ultra-short-acting opioid Remifentanil as a co-analgesic.^37^ The remaining nine trials were more homogenous, essentially comparing Remimazolam infusions with Propofol in East Asian populations while using short-acting synthetic opioids like Fentanyl or Alfentanil. This concentration of heterogeneity within two studies with unique protocols suggests that while our overall pooled estimates are valuable, the clinical effects of Remimazolam are significantly affected by the choice of comparator drug, dosing strategy, patient ethnicity, and the specific opioid used.

Another significant limitation stems from the geographic concentration of most included RCTs. Since most of them were conducted in China, the ethnic diversity of the patients is narrow, which raises clear questions about generalizability. This is a notable consideration for Remimazolam, as it is metabolized by tissue Carboxylesterases (CES). Genetic variations in these enzymes are common within the Chinese population, and these polymorphisms can significantly affect how a person responds to a drug, affecting both its efficacy and potential for toxicity.^43^^,^^44^ Because these genetic profiles differ across the globe, the specific safety and effectiveness we observed might not be the same for patients in North America or Europe.^45^ Therefore, validating our findings in trials with more diverse, multi-ethnic populations is a necessary next step to truly understand Remimazolam's global clinical value. Furthermore, in two of the studies included in this analysis, flumazenil was routinely administered at the end of the procedure to reverse sedation.^23^^,^^25^ This practice was standard protocol rather than a response to adverse events and could potentially have influenced outcomes such as recovery time and patient satisfaction. However, including these studies did not increase heterogeneity or change the overall significance of the pooled results, including those related to recovery time and satisfaction.

Further methodological limitations should also be acknowledged. Data regarding three outcomes, time to recovery of consciousness, induction time, and patient satisfaction, were reported as median and IQR or median and range. Despite applying the validated methods proposed by Luo and Wan, this variability can lead to inaccuracies and imprecision.^28^^,^^29^ Additionally, the precision of our estimates in certain subgroup analyses was constrained by the limited number of included trials. This scarcity of head-to-head comparisons, combined with the overall clinical heterogeneity, ultimately precluded our ability to perform a meta-regression to investigate the dose-related effects of Remimazolam.

## Conclusion

Remimazolam is associated with a significantly lower risk of hypotension, bradycardia, hypoxia, and respiratory depression compared to pooled sedatives. Our findings clarify the clinical trade-offs: it is demonstrably safer than Propofol, while also offering greater sedation success than Midazolam, higher satisfaction, lower hypoxia incidence, shorter induction time and faster recovery than Dexmedetomidine. It presents a reliable balance of safety and efficacy, making it a valuable agent for FB. Nonetheless, these conclusions should be interpreted in light of certain methodological limitations, such as the presence of high heterogeneity in some outcomes, the predominance of single-country data, and the limited number of trials for specific subgroup comparisons. Future trials should focus on standardized dosing, vulnerable populations, and the pharmacoeconomic impact of a broad adoption of Remimazolam.

## Data availability statement

The datasets generated and/or analyzed during the current study are available from the corresponding author upon reasonable request.

## Abbreviations

ASA, American Society of Anesthesiology; BPM, Beats Per Minute; FB, Flexible Bronchoscopy; GABA_A_, γ-Aminobutyric Acid A; HR, Heart Rate; IQR, Interquartile Range; MAP, Mean Arterial Pressure; MD, Mean Difference; PRISMA, Preferred Reporting Items for Systematic Reviews and Meta-Analysis; PROSPERO, Prospective Register of Systematic Reviews; RCT, Randomized Controlled Trial; RR, Risk Ratio; SBP, Systolic Blood Pressure; SD, Standard Deviation; SMD, Standard Mean Difference; USA, United States of America.

## Artificial Intelligence (AI) statement

No AI tools were used in this manuscript.

## Authors’ contributions

Luiz Fábio Silva Ribeiro: Conceptualization; Writing-original draft; Project administration, Lucas Rezende de Freitas: Conceptualization; Methodology; Writing-original draft; Validation; Project administration, Tauãna Terra Cordeiro de Oliveira: Formal analysis; Investigation; Validation, Laiz Gomes Carneiro Novaes: Formal analysis; Investigation; Validation, Rafael Arsky Lombardi: Supervision; Validation; Writing-review & editing.

All authors read and approved the final version of the manuscript.

## Funding

This research received no specific grant from funding agencies in the public, commercial, or not-for-profit sectors.

## Declaration of competing interest

The authors certify no conflict of interest with any financial organization regarding the material discussed in the manuscript.

## Appendix Supplementary materials

Image, application 1

## Footnotes

Supplementary material associated with this article can be found in the online version at doi:10.1016/j.bjane.2026.844729.
