# Supplementary material for: Remimazolam compared with propofol, dexmedetomidine, and midazolam for adult sedation in flexible bronchoscopy: a systematic review and meta-analysis
Source: Braz J Anesthesiol. 2026 Jan 24;76(3):844729. doi: 10.1016/j.bjane.2026.844729 (PMC12961330; doi:10.1016/j.bjane.2026.844729)
Supplement: Supplementary file 1 [file mmc1.docx]

**BJAN-D-25-00337_ Supplementary Material**

**Supplemental Figure** **1** (A) Leave-one-out sensitivity analysis of hypotension. (B) Leave-one-out sensitivity analysis of bradycardia. (C) Leave-one-out sensitivity analysis of intraprocedural opioid consumption. (D) Leave-one-out sensitivity analysis of respiratory depression. (E) Leave-one-out sensitivity analysis of patient satisfaction score. (F) Leave-one-out sensitivity analysis of hypoxia. (G) Leave-one-out sensitivity analysis of induction time. (H) Leave-one-out sensitivity analysis of success of sedation. (I) Leave-one-out sensitivity analysis of time to complete recovery of consciousness.


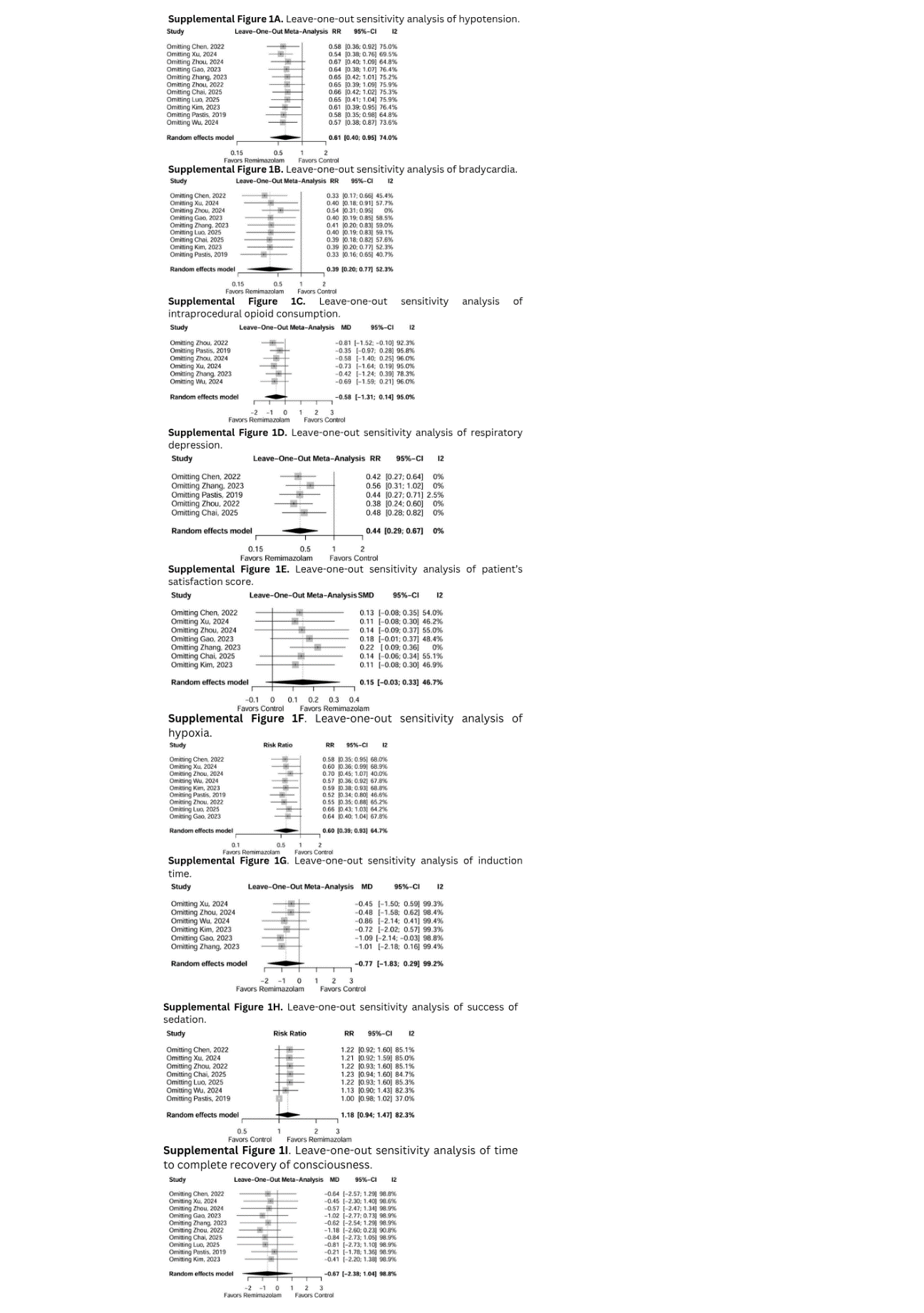


**Supplemental Figure 2** Leave-one-out sensitivity analysis of hypotension.


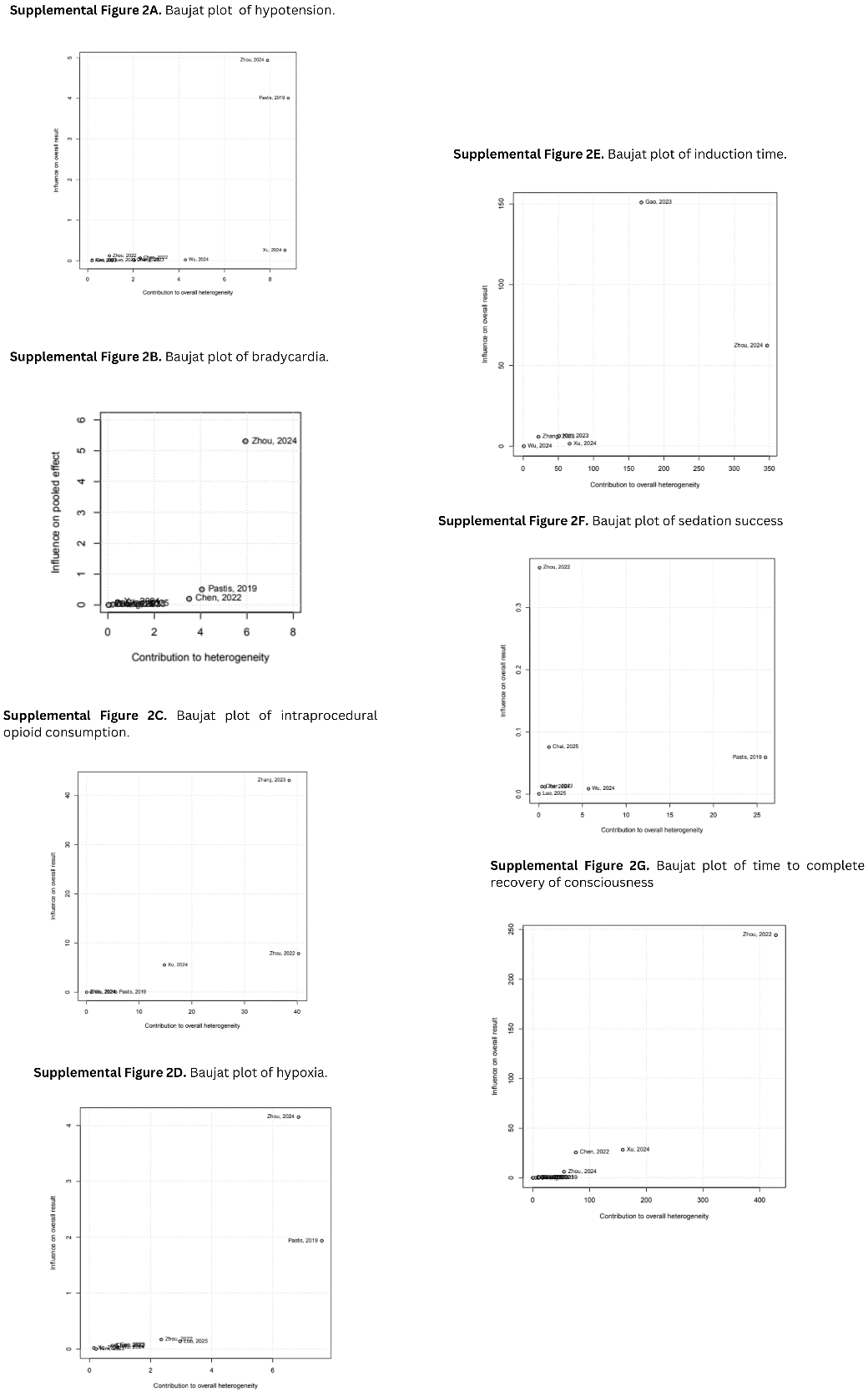


**Supplemental Figure 3** (A) Funnel plot and Egger’s test for hypotension. (B) Funnel plot and Egger’s test for time to complete recovery of consciousness.

**Supplementary Appendix** Exact search strategy performed in PubMed, Embase and Cochrane databases: (Remimazolam OR Byfavo OR “CNS 7056”).
